# Identification and Characterization of Microcin S, a New Antibacterial Peptide Produced by Probiotic *Escherichia coli* G3/10

**DOI:** 10.1371/journal.pone.0033351

**Published:** 2012-03-30

**Authors:** Anke Zschüttig, Kurt Zimmermann, Jochen Blom, Alexander Goesmann, Christoph Pöhlmann, Florian Gunzer

**Affiliations:** 1 Institute of Medical Microbiology and Hygiene, TU Dresden, Dresden, Germany; 2 SymbioPharm GmbH, Herborn-Hörbach, Germany; 3 CeBiTec, University of Bielefeld, Bielefeld, Germany; 4 Abteilung für Labormedizin, Robert Bosch Krankenhaus, Stuttgart, Germany; Charité-University Medicine Berlin, Germany

## Abstract

*Escherichia coli* G3/10 is a component of the probiotic drug Symbioflor 2. In an *in vitro* assay with human intestinal epithelial cells, *E. coli* G3/10 is capable of suppressing adherence of enteropathogenic *E. coli* E2348/69. In this study, we demonstrate that a completely novel class II microcin, produced by probiotic *E. coli* G3/10, is responsible for this behavior. We named this antibacterial peptide microcin S (MccS). Microcin S is coded on a 50.6 kb megaplasmid of *E. coli* G3/10, which we have completely sequenced and annotated. The microcin S operon is about 4.7 kb in size and is comprised of four genes. Subcloning of the genes and gene fragments followed by gene expression experiments enabled us to functionally characterize all members of this operon, and to clearly identify the nucleotide sequences encoding the microcin itself (*mcsS*), its transport apparatus and the gene *mcsI* conferring self immunity against microcin S. Overexpression of cloned *mcsI* antagonizes MccS activity, thus protecting indicator strain *E. coli* E2348/69 in the *in vitro* adherence assay. Moreover, growth of *E. coli* transformed with a plasmid containing *mcsS* under control of an *araC* PBAD activator*-*promoter is inhibited upon *mcsS* induction. Our data provide further mechanistic insight into the probiotic behavior of *E. coli* G3/10.

## Introduction

Microcins are ribosomally synthesized antimicrobial peptides with a low molecular mass. Produced by enterobacteria, mostly *Escherichia coli*, microcin synthesis is sharply activated under stress conditions such as limitation of nutrients [1]–[4]. Microcins exert potent antibacterial activity against closely related species, which offers a highly competitive advantage in the intestinal microflora [5]. Microcin-producers are resistant to the microcin they produce, which is mediated by at least one resistance-conferring gene located within one gene cluster. Most of the 14 known microcins are plasmid-encoded [5]–[10], but chromosomally-encoded antibacterial peptides have also been described [11]. The probiotic strain *E. coli* Nissle 1917 (EcN) is known to produce microcins M and H47 [12]. In this study we show that probiotic *E. coli* G3/10 produces a novel microcin, that we named microcin S (MccS). *E. coli* G3/10 is one of six *E. coli* genomotypes present in the probiotic drug Symbioflor 2 (DSM17252). the product has been successfully used for the treatment of functional gastrointestinal disorders, in particular irritable bowel syndrome in adults and children [13], [14]. Probiotics are defined as living microorganisms, which upon ingestion in certain numbers, exert health benefits beyond inherent basic nutrition [15]. The mechanisms that enable a strain to serve as a probiotic are poorly understood. Nevertheless, the antimicrobial activity of microcins could positively influence the stability of the intestinal microflora. Given its extensive clinical safety record, a microcin-producing strain containing no virulence factors can clearly fulfill the definition of a probiotic. In contrast to enterobacterial microcins, food-borne lactic acid bacteria produce lanthionine-containing peptide antibiotics. The so-called lantibiotics of gram-positive bacteria are already used for food preservation [16]. The use as an antitumor agent [17] or as an alternative to classical antibiotics in infectious diseases [18], [19] are two further applications where bacteriocins may provide therapeutic alternatives in the future. The worldwide emergence of pathogens resistant to antibiotics has led to an ever-increasing demand of new antibacterial agents. Enterobacterial microcins could offer exciting new possibilities for prophylaxis and treatment of bacterial infections. Here we present the identification and functional characterization of microcin S, a completely novel plasmid encoded bacteriocin produced by probiotic *E. coli* G3/10. Microcin S is able to inhibit the adherence of enteropathogenic *E. coli* (EPEC) strain E2348/69 to intestinal epithelial cells in an *in vitro* adherence assay and growth of *E. coli* is hampered by L-arabinose induced recombinant expression of MccS.

**Figure 1:**
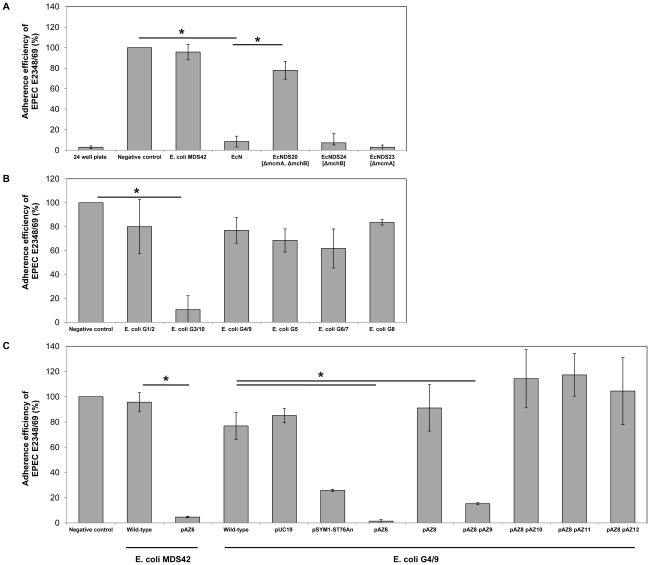
Adherence efficiency of EPEC E2348/69 to human intestinal epithelial cells after pre-incubation with *E. coli* Nissle 1917 (EcN) and EcN deletion strains (EcNDS) (A); *E. coli* G1/2, *E. coli* G3/10, *E. coli* G4/9, *E. coli *G5, *E. coli* G6/7 and *E. coli* G8, the components of Symbioflor 2 (DSM17252) (B); *E. coli* strains MDS42 and G4/9 wild-type and appropriate mutants (C). Confluent monolayers of CACO-2 or LOVO cells were pre-incubated with bacterial test strains at an MOI of 100∶1 *E. coli* to host cells. After two hours of incubation, cells were washed and infected with EPEC E2348/69 using an MOI of 100∶1 EPEC to host cells. Adherence efficiency in % is expressed as adherence of EPEC relative to the adherence without any pre-incubation (negative control), which is set at 100%. Pre-incubation of human intestinal epithelial cells with EcN significantly reduces adherence efficiency of EPEC E2348/69, comparable to the mutant strains EcNDS24 and EcNDS23 which carry single genomic deletions of either microcin H47 precursor (*mchB*; EcNDS24) or microcin M precursor (*mcmA*; EcNDS23). However, this phenomenon cannot be observed, when double microcin mutant EcNDS20 is used (A). The adherence efficiency of EPEC E2348/69 is also significantly reduced in the presence of microcin S, either expressed by *E. coli* G3/10 wild-type (B) or by *E. coli* strains MDS42 and G4/9, transformed with plasmids pSYM1-ST76An (*mcsS, mcsI, mcsA, mcsB*), pAZ6 (*mcsS, mcsI, mcsA, mcsB*) or pAZ9 (*mcsS*) (C). With all further plasmids, pAZ8 (*mcsI, mcsA, mcsB*), pAZ10 (ORF1), pAZ11 (ORF2) and pAZ12 (*mcsS*_193–363_), the indicator strains *E. coli* MDS42 and G4/9 did not inhibit adherence of EPEC E2348/69 to intestinal epithelial cells (C). Data are the mean±SD of at least three separate experiments in duplicate wells. * p≤0.01 compared to negative controls or wild-type strains.

## Results

### Evaluation of the In vitro Adherence Assay

Bacterial adhesion is a crucial first step of many infectious diseases. Therefore, a test system quantifying adherence inhibition of enteropathogens to human intestinal epithelial cells is a suitable model system to evaluate this beneficial effect to the host. Initially, we used enteropathogenic *E. coli* strain E2348/69 [20] to investigate adherence efficiency to human intestinal epithelial cells (LOVO or CACO-2) in response to a pre-incubation with different probiotic or non-probiotic *E. coli* isolates. We demonstrated that EcN significantly inhibits EPEC adherence (Fig. 1A), which is consistent with the results of Kleta et al. [21] who used a similar assay with porcine intestinal IPEC-J2 cells. Since EcN produces two different microcins M and H47 [12], we assumed that the observed effect resulted from those antibacterial peptides, which can inhibit bacterial growth and kill target bacteria. Indeed, we were able to show that an EcN deletion strain (EcNDS20), negative for both microcins M and H47, is not able to inhibit EPEC adhesion (Fig. 1A), whereas deletion of only one microcin (EcNDS23 and EcNDS24) resulted in wild-type behavior (Fig. 1A). Therefore, the *in vitro* adherence assay used here also provides a suitable test environment for detecting bactericidal activity of a given strain specifically directed against the used adherent strain.

**Figure 2:**
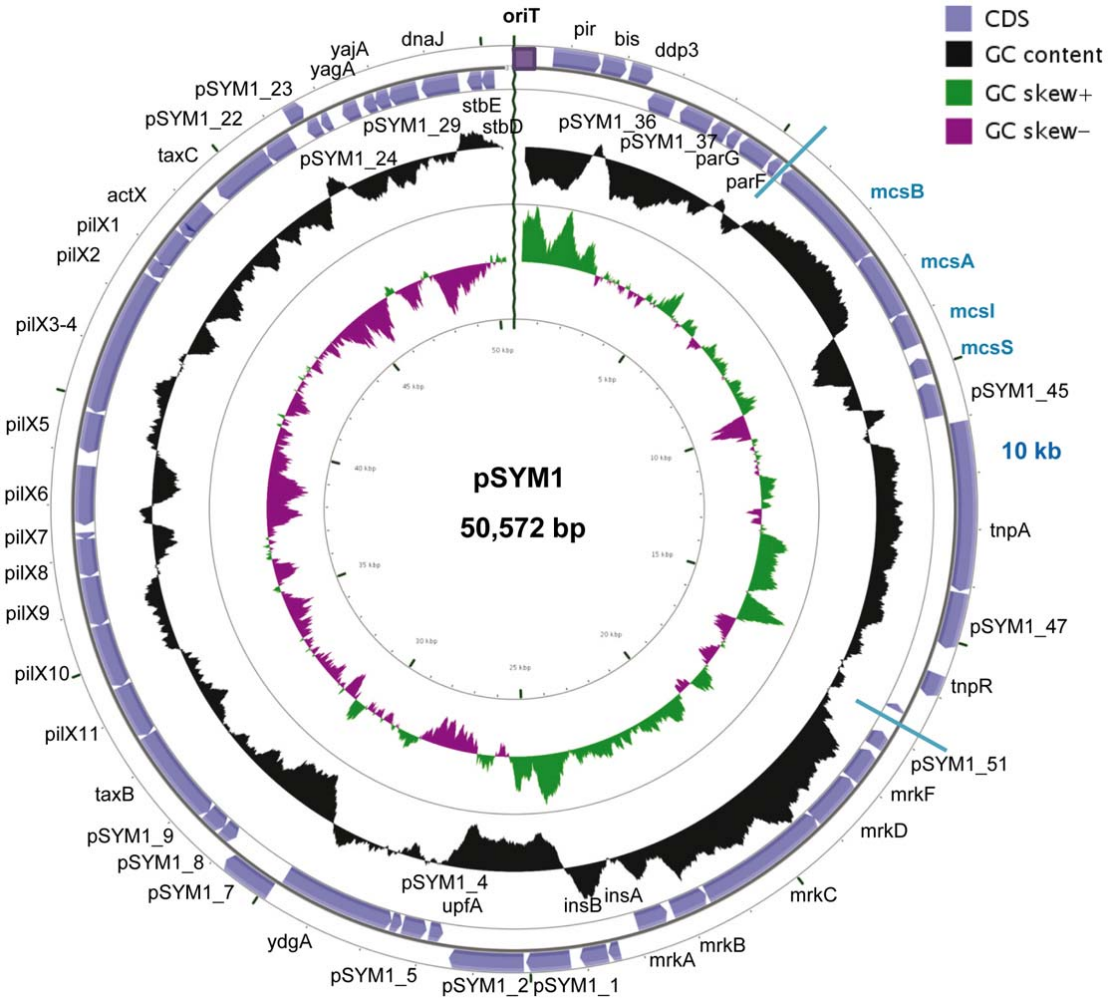
Circular diagram of megaplasmid pSYM1. The outer circle indicates (to scale) the genetic organization of ORFs within the plasmid. The direction of transcription of each ORF is indicated. The middle circle indicates the GC content, and the inner circle indicates the GC skew. The 10 kb region of pSYM1 that is different from pMAS2027 is marked. The image was constructed using cgview [41]. CDS = coding sequences.

### Identification of the Microcin S-Encoding Gene Cluster in E. coli G3/10

**Figure 3:**
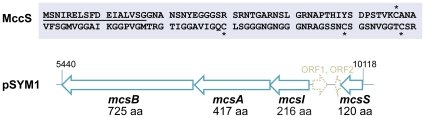
The MccS gene cluster. The upper panel shows the amino acid sequence of the microcin S precursor. A probable leader peptide is underlined. Asterisks indicate cysteines possibly involved in the formation of a disulfide bond typical for class II microcins. The microcin S gene cluster on megaplasmid pSYM1 (lower panel) consists of the four clustered genes mcsS, mcsI, mcsA and mcsB. ORF1 and ORF2 revealed no bactericidal activity.

The EPEC *in vitro* adherence assay was then repeated with all genomotypes of the probiotic drug Symbioflor 2, which are *E. coli* G1/2, G3/10, G4/9, G5, G6/7 and G8. Surprisingly, we could show that *E. coli* G3/10 also significantly inhibited EPEC adherence efficiency (Fig. 1B) indicating a bactericidal activity of that strain. The genome of *E. coli* G3/10 was sequenced in our laboratory (unpublished data). However, neither during manual editing of the automatically annotated sequence nor with a BLAST analysis, could any coding sequences of a known microcin be identified within the genome of *E. coli* G3/10. Microcins are often encoded by plasmids [5]. *E. coli* G3/10 contains a large conjugative plasmid pSYM1 (Fig. 2), having a size of 50.6 kb. The plasmid is 99% identical to plasmid pMAS2027 of uropathogenic *E. coli* MS2027 [22]. However, it additionally contains a 10 kb insertion fragment, which carries only uncharacterized and unnamed genes. To identify the origin of *E. coli* G3/10s bactericidal action we first tried to cure the strain from its megaplasmid pSYM1. However, while using several common curing procedures such as treatment with mitomycin C or heat, we failed to remove pSYM1 from *E. coli* G3/10. Therefore, the plasmid was transferred to *E. coli* G4/9 by conjugation. To allow screening of conjugants, we first integrated an ampicillin resistance cassette into pSYM1 resulting in pSYM1-ST76An (Table 1). *E. coli* G4/9 transformed with pSYM1-ST76An was able to inhibit EPEC adherence significantly (Fig. 1C). It logically followed that plasmid pSYM1 carries genes responsible for the observed effect. Then, we cloned a 4.7 kb subfragment of plasmid pSYM1 into pBR322, resulting in plasmid pAZ6 (Table 1), which was subsequently transformed into *E. coli* G4/9. We demonstrated that pAZ6 enables *E. coli* G4/9 to inhibit EPEC adherence efficiency significantly (Fig. 1C), indicating that the 4.7 kb fragment of pSYM1 (Fig. 3) is responsible for the EPEC adherence inhibition effect of *E. coli* G3/10. BLAST analysis of automatically annotated open reading frames (ORFs) in this segment of pSYM1 revealed small homologies to characterized proteins or protein families belonging to microcin-encoding operons. Nevertheless, the microcin itself remained undetected. For this reason, we cloned three genes, named *mcsI*, *mcsA* and *mcsB* in the following, into pBR322 leading to pAZ8 (Table 1). Small ORFs upstream of this operon (Fig. 3), which were candidate microcin-encoding genes, were cloned into pACYC184 and subcloned into *E. coli* G4/9 pAZ8. With this strategy we successfully managed a sequential plasmid-based identification and characterization of potential microcin-coding regions together with the respective microcin-helper proteins. Only *E. coli* G4/9, containing plasmids pAZ8 and pAZ9 (Table 1) together, significantly inhibited EPEC adherence, whereas *E. coli* G4/9 pAZ8 affected EPEC adherence similar to that of *E. coli* G4/9 wild-type (Fig. 1C). We therefore concluded that the small gene cloned into pAZ9, which we termed *mcsS*, encodes a novel *E. coli* microcin, named microcin S in the following. Truncated *mcsS* (pAZ12; Table 1) resulting from an alternative ORF did not show microcin activity in the *in vitro* adherence assay (Fig. 1C).

**Table 1:** Plasmids used in this study.

Plasmid	Resistance	Relevant genotype	Origin
pSYM1	None	*mcsS, mcsI, mcsA, mcsB*	This work
pST76–A	Amp^r^	ori^TS (30°C)^	[43]
pSYM1–ST76An	Amp^r^	*mcsS, mcsI, mcsA, mcsB*	This work
pAZ6	Amp^r^	*mcsS, mcsI, mcsA, mcsB*	This work
pAZ8	Amp^r^	*mcsI, mcsA, mcsB*	This work
pAZ9	Cm^r^	*mcsS*	This work
pAZ10	Cm^r^	ORF1	This work
pAZ11	Cm^r^	ORF2	This work
pAZ12	Cm^r^	*mcsS*_193–363_	This work
pAZ13	Amp^r^	*mcsI*	This work
pAZ14	Amp^r^	*mcsI*_361–651_	This work
pGS1	Amp^r^	PBAD	This work
pAZ15	Amp^r^	PBAD, *mcsS*	This work

Amp^r^ = ampicillin resistance; Cm^r^ = chloramphenicol resistance; ORF = open reading frame; PBAD = araC PBAD activator-promoter.

### Microcin S Immunity

**Figure 4:**
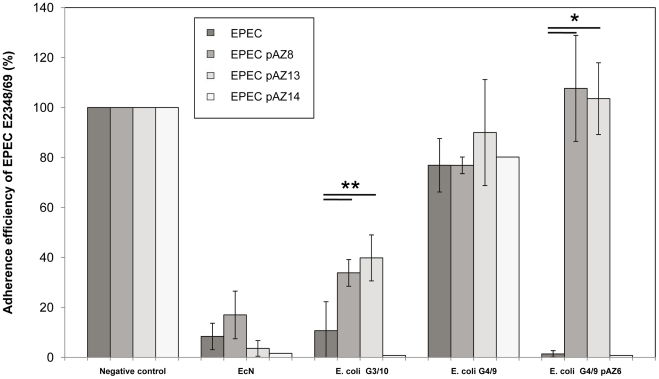
Adherence efficiency of wild-type and mutant strains of EPEC E2348/69 to human intestinal epithelial cells after pre-incubation with different microcin positive and negative *E. coli* strains. Confluent monolayers of CACO-2 or LOVO cells were pre-incubated with bacterial test strains EcN, E. coli G3/10, microcin-negative E. coli G4/9 wild-type or microcin S expressing E. coli G4/9 pAZ6 at an MOI of 100∶1 E. coli to host cells. After two hours of incubation, cells were washed and infected with EPEC E2348/69 using an MOI of 100∶1 EPEC to host cells. Adherence efficiency in % is expressed as adherence of EPEC relative to the adherence without any pre-incubation (negative control), which is set at 100%. In EPEC E2348/69 plasmids pAZ8 (mcsI, mcsA, mcsB) and pAZ13 (mcsI) containing full-length gene mcsI confer immunity to mcsS expressing strains E. coli G3/10 and E. coli G4/9 pAZ6. However, EPEC indicator strain transformed with pAZ14 carrying truncated mcsI361-651 is not resistant to microcin S. None of the plasmids used procures immunity against EcN microcins H47 and M. Data are the mean±SD of at least three separate experiments in duplicate wells. * p ≤ 0.01, ** p ≤ 0.05.

Class II microcins are characterized by a dedicated self-immunity protein. Gene *mcsI* adjacent to *mcsS* shows homology to the CAAX amino terminal protease family potentially involved in microcin self-immunity. No signal sequence or transmembrane helices are predicted. We transformed *mcsI* into EPEC strain E2348/69 using either pAZ8 (*mcsI, mcsA, mcsB*), pAZ13 (*mcsI *) or pAZ14 (*mcsI*, truncated). We could show that the EPEC strain carrying the complete *mcsI* gene (pAZ8 or pAZ13) is resistant to microcin S-producing *E. coli* G3/10 or *E. coli* G4/9 pAZ6 in the *in vitro* adherence assay. Truncated *mcsI* (pAZ14) was not able to confer microcin S resistance. Therefore we identified gene *mscI*, which encodes a 216 amino acid protein, as responsible for microcin S self-immunity. Additionally, we demonstrated that *mcsI* is not effective against EcN microcins M and H47 (Fig. 4).

### Investigation of Microcin S Activity in E. coli MDS42

**Figure 5:**
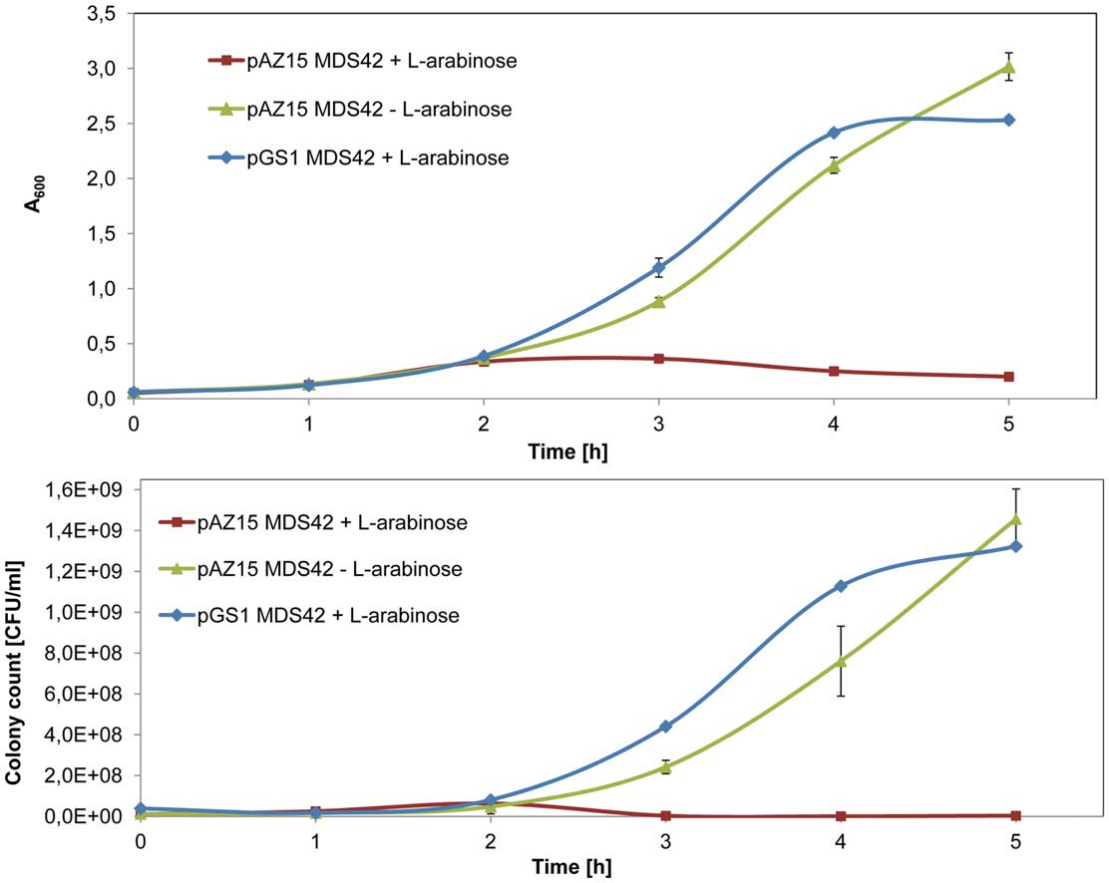
Growth curve of *E. coli* MDS42 with pAZ15 or pGS1 showing A600(A) and colony count of viable cells (B). pAZ15 is a vector containing mcsS under control of the L-arabinose-induced araC PBAD activator-promoter. Induction with L-arabinose of the respective cultures, as indicated in the legend to panel A and B, was carried out after 90 minutes. pGS1 is the empty vector with the araC PBAD activator-promoter. E. coli MDS42 with pAZ15 shows a significant reduction of its A600 as well as of its colony counts after induction with L-arabinose (red squares) compared to growth without induction (green triangles) and also with the vector devoid of mcsS (pGS1, blue rhombi). Data are the mean±SD of three independent experiments.

In order to directly demonstrate microcin S activity against a susceptible *E. coli* strain, *mcsS* was cloned into pGS1 resulting in the plasmid pAZ15 (Table 1). In this construct, *mcsS* expression is controlled by an *araC* PBAD activator*-*promoter, rendering microcin S expression inducible by L-arabinose. When *E coli* MDS42 [23] growing in liquid culture after being transformed with pAZ15 was treated with 0.2% v/v L-arabinose at the beginning of the logarithmic phase after 1.5 h, A_600_ remained almost stable around 0.3 while absorbance of control cultures increased constantly (Fig. 5A). Counts of colony forming units (CFU) taken at various time points during this experiment revealed a sharp drop in the number of viable bacteria in the L-arabinose induced culture of *E. coli* MDS42+pAZ15 while CFU of the other cultures rose to a maximum of 1.4 × 10^9^/ ml (Fig. 5B).

### Presence of Microcin S Gene in Enterobacteriaceae

**Figure 6:**
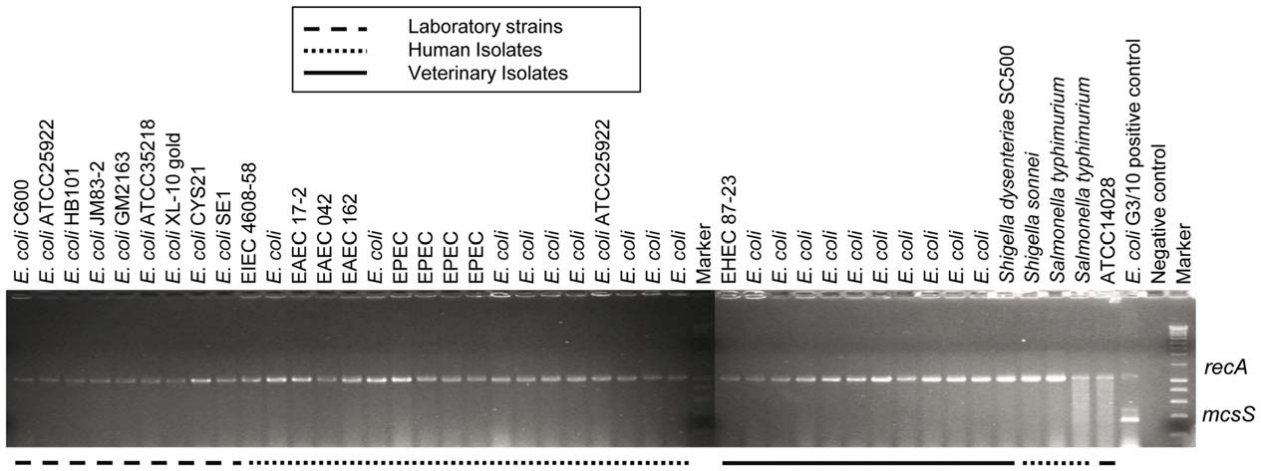
Multiplex PCR to screen for presence of *mcsS* using *recA* as inhibition control. The tested strains are common laboratory strains, human clinical isolates and veterinary isolates of different origin. *mcsS* could not be detected in any of the tested strains while the amplified inhibition control indicated that all PCR reactions were truly negative.

The sequences of *mcsS* and *mcsI* were listed very recently (04.09.2011) as new entries in the NCBI database, being part of the 54 kb megaplasmid pMO17_54 (accession # HE578057) of *Shigella* sp. MO17. The proteins derived from these coding sequences, termed pMO17_54_21 and pMO17_54_23, were described as hypothetical proteins and no function was assigned to them. Nevertheless, a complete MccS operon is not listed in NCBI (as of 21.10.2011). A further 38 *E.* *coli,* two Shigella and two Salmonella strains were screened for *mcsS* using a multiplex PCR protocol. We were unable to detect the *mcsS* gene in any of the 42 human and veterinary isolates or the laboratory strains tested (Fig. 6).

**Table 2:** Bacterial strains used in this study.

Strain	Relevant genotype	Origin
*E. coli* Nissle 1917 DSM6601 [42]	Wild-type	Mutaflor, Ardeypharm
EcNDS20	*?mcmA*, *?mchB*	This work
EcNDS23	*?mcmA*	This work
EcNDS24	*?mchB*	This work
*E. coli* G1/2	Wild-type	Symbioflor 2^a^, SymbioPharm
*E. coli* G3/10	Wild-type	Symbioflor 2^a^, SymbioPharm
*E. coli* G4/9	Wild-type	Symbioflor 2^a^, SymbioPharm
*E. coli* G5	Wild-type	Symbioflor 2^a^, SymbioPharm
*E. coli* G6/7	-type Wild	Symbioflor 2^a^, SymbioPharm
*E. coli* G8	Wild-type	Symbioflor 2^a^, SymbioPharm
*E. coli* MDS42 [23]	*E. coli* K-12 multiple deletion strain	F. Blattner, University of Wisconsin - Madison, USA
EPEC E2348/69 [20]	Amp^r^ (pUC19), Kana^r^ (pUC4k) or Cm^r^ (pACYC184)	Human isolate

a DSM17252; EcNDS = E. coli Nissle 1917 deletion strain; Amp^r^ = ampicillin resistance; Kana^r^ = kanamycin resistance; Cm^r^ = chloramphenicol resistance.

**Table 3:** Oligonucleotide primers used in this study.

Oligonucleotide	Sequence (5′→ 3′)	Function
mcm-H1	cttaaagcgttacataggcaccattatcatataatgaagcaccgattgtgtaggctggagctgcttc	EcN deletion primer
mcm-H2	gaatttttacttcttcacaaatcttatagcgaaggtgttgaaatggtccatatgaatatcctcctta	EcN deletion primer
mch-H1	atcaacgactgtaaatcatatcttcatcagtaaagtgttgaacgattgtgtaggctggagctgcttc	EcN deletion primer
mch-H2	ggtcaggctggaaaaacggaagttaaatatgatggagtttatatggtccatatgaatatcctcctta	EcN deletion primer
mcm_for	cgttcggaggagcctaac	EcN deletion primer/sequencing
mcm_rev	gattcatgggattcgaagg	EcN deletion primer/sequencing
Contig49_for	cagctggatatcctgcgcg	pSYM1 sequencing primer
Contig49_rev	ggttgcccggcatccaacg	pSYM1 sequencing primer
pSYM1-SalIHF	tcaattgtgtcgactcaattactcttgtgag	pAZ6/pAZ8 cloning primer
pSYM1-NheI	catgtaatagtgctagcatgttaaaatttataag	pAZ6 cloning primer
pSYM1-NheIac	caaaaataatagctagcaagtgatgttttgtaatg	pAZ8, pAZ12 cloning primer
ac-EcoRI	ctcgaattcatccattacaaaacatcac	pAZ9 cloning primer
ac-PstI	ctggctgcagtaattgttcaggaagtaacg	pAZ9 cloning primer
pSYM1_44–EcoRI	taggaattcagaggaactattggtggg	pAZ10 cloning primer
pSYM1_44–PstI	ctccgctgcagacttacttatcgactacaggtaccac	pAZ10 cloning primer
ab-EcoRI	gttagaattcataagagggatttttatgtcaaatatc	pAZ11 cloning primer
ab-PstI	gttgatactgcagcttatcgactacaggtaccacc	pAZ11 cloning primer
pAZ9-HindIII	cccaagcttagttaaatgtgctaatgctgtc	pAZ12 cloning primer
pAZ9-SalI	ggcatcggtcgacgcaac	pAZ12 cloning primer
pSYM1_43–NheI	cattgctagccatcacagataaactggataac	pAZ14 cloning primer
pSYM1_43–SalI	ccctgagtcgactcatggttataaaatattttg	pAZ13, pAZ14 cloning primer
recA–ff	atggctatcgacgaaaacaaac	Multiplex PCR inhibition control
recA–rev	ttaaaaatcttcgttagtttctgc	Multiplex PCR inhibition control
mcsS–ff	atgtcaaatatcagagaattgag	mcsS PCR screening primer
mcsS–rev	ttatcgactacaggtaccacc	mcsS PCR screening primer

Oligonucleotides were synthesized by Biomers, Ulm, Germany.

## Discussion

Microcins are small ribosomally synthesized peptides produced by enterobacteria. We could show that *E. coli* G3/10, an *E. coli* genomotype of Symbioflor 2 (DSM17252), encodes and produces a completely novel class II microcin, named microcin S. The term microcin was introduced to distinguish this class of antibacterial peptides with a size <10 kDa from colicins with a higher molecular mass [24]. Genome sequencing of *E. coli* G3/10 enabled us to indentify the gene *mcsS*, located on plasmid pSYM1, encoding the 120 amino acid (aa) microcin S precursor peptide. It has a calculated molecular weight of 11.67 kDa and is therefore the largest of all currently known microcins. Using the Pfam database [25] microcin S can be classified as a class II bacteriocin with a double-glycine leader peptide. Indeed, its amino acid sequence reveals a glycine-rich peptide with a double-glycine cleavage site and cysteines probably involved in formation of a disulfide bond (Fig. 3). These characteristics, together with the organization of the microcin gene cluster (Fig. 3), indicate MccS to be a member of the class IIa microcins. The current classification follows a discussion of Duquesne et al. [26] and Rebuffat [27]. MccV (formerly colicin V), MccL and Mcc24 are three members assigned to the microcin class IIa. All are composed of four plasmid-borne genes, mostly harbored by large conjugative low-copy-number plasmids [7], [10]. The genes for MccV and MccL, *cvaC* and *mclC*, encode 103 and 105 aa precursors, which also constitute higher-molecular mass microcins. Both peptides form disulfide bonds [28], [29]. Class IIa microcins have leader peptides whose cleavage results in the mature biologically active peptide. Furthermore, they are not subject to post-translational modification. Gene clusters of all class IIa microcins consist of four genes organized in one or two transcription units: the gene encoding the microcin precursor, a self-immunity protein, an accessory protein involved in the secretion of the microcin, and an ATP-binding cassette (ABC)-transporter (for reviews see [26], [30]). The self-immunity genes code for small peptides that protect the producing strain from its own microcin. Here we can show that gene *mcsI* cloned into EPEC E2348/69 confers immunity to MccS. Gene *mcsI* encodes a 216 aa protein of the CAAX amino terminal protease protein family. The two genes comprising the MccS export system are *mcsA* and *mcsB* (Fig. 3). Gene *mcsA* is a member of the *E. coli* HlyD family and shows little homology to the colicin secretion protein CvaA. Gene *mcsB* encodes an ABC-transporter consisting of a transmembrane domain, an ATP-binding domain and a peptidase domain [25]. The nucleotide lengths of *mcsA* and *mcsB* from the MccS operon, 1254 bp and 2178 bp respectively, are comparable to export genes of the other microcins MccV (*cvaA*, 1242 bp; *cvaB*, 2097 bp), MccL (*mclA*, 1242 bp; *mclB*, 2097 bp) and Mcc24 (*mtfA*, 1245 bp; *mtfB*, 2124 bp) [26]. The sensitivity of microorganisms to microcins can generally be shown by a standard agar diffusion test, for example with the microcin-producing strain spotted onto an agar plate and an indicator strain inoculated into a soft agar overlay [12]. However, *E. coli* G3/10 does not produce real inhibition zones in agar diffusion tests (data not shown). This phenomenon can possibly be explained by the widely unknown conditions for effective MccS expression. Motivated by the fact that synthesis of MccV is repressed by excess iron [31], we added the iron chelator Desferal to the solid media growing *E. coli* G3/10 in the diffusion test. However, microcin production on agar plates could still not be shown. We therefore assume that other factors, such as pH or nutrients, may be involved in the regulation of microcin S expression. Because the adherence assay provides only indirect proof of the antibacterial activity of MccS-producing *E. coli* strains and the above mentioned agar diffusion test did not work properly with microcin S, we decided to subclone *mcsS* under the control of an *araC* PBAD activator-promoter. When microcin S production was induced with L-arabinose in *E. coli* MDS42 growing in liquid culture, viable counts dropped by several logs, clearly indicating a direct toxic activity of MccS against a susceptible *E. coli* strain. MccS from *E. coli* G3/10 has the great advantage that the microcin does not necessarily have to be purified from the strain. Since *E. coli* G3/10 is set out in a probiotic formulation that can be used for treatment of gastrointestinal disorders, MccS is very much likely to also be expressed effectively *in vivo*, rendering this beneficial microorganism a promising biological drug with prophylactic capacities against enteric pathogens such as Salmonella, Shigella or diarrheagenic *E. coli*.

## Materials and Methods

### Bacterial Strains and Growth Conditions

Bacterial strains used in this work are listed in table 2. Bacteria were grown in Luria Bertani (LB) medium or on LB agar plates (Becton Dickinson, BD, Heidelberg, Germany). Culture media were supplemented with ampicillin (100 µg ml^–1^), chloramphenicol (25 µg ml^–1^), kanamycin (50 µg ml^–1^), tetracyclin (12.5 µg ml^–1^) or streptomycin (30 µg ml^–1^) as required. Antibiotics were purchased from AppliChem, Darmstadt, Germany.

### Construction of EcN Microcin Deletion Mutants

EcN mutants negative for chromosomally encoded microcins M and H47 (*mcmA, mchB*) were constructed with a PCR-based one-step inactivation method [32], [33]. Deletions were controlled by PCR using primer pairs outlined in table 3 as well as automated DNA sequencing based on dideoxy chain termination method [34], using an ABI 3100 sequencing instrument (Life Technologies, Darmstadt, Germany).

### Construction of Plasmids Used for Functional Characterization of the Microcin S Operon

The targeted sequence was amplified by PCR using oligonucleotides with 5′ restriction sites (Table 3). Due to their proofreading capacity, PCR was performed with Phusion High-Fidelity DNA-Polymerase (Thermo Fisher Scientific/Finnzymes, Vantaa, Finland) according to the recommended protocol. Following purification of PCR products with columns containing silica membranes (Qiagen, Hilden, Germany), fragments were digested with the respective restriction enzymes (New England Biolabs, NEB, Frankfurt (Main), Germany) and ligated into an appropriate vector using T4 DNA-Ligase (NEB). Ligated DNA constructs were then transformed to competent bacteria by electroporation (2.5 kV; 400 Ω; 25 µFD) using a Bio-Rad Gene Pulser II and Pulse Controller Plus (Munich, Germany). Clones were selected on LB agar plates containing the appropriate antibiotic and were confirmed by PCR, DNA sequencing and by restriction digest using the respective enzymes.

### Conjugation Experiments

The conjugative plasmid pSYM1 allows the transfer to a recipient strain. pSYM1 was mobilized from *E. coli* G3/10 into *E. coli* G4/9 following a protocol published by Miller et al. [35] with slight modifications. *E. coli* G4/9 was chosen as the recipient strain because it is closely related to *E. coli* G3/10 and harbors only one natural plasmid (unpublished data). Since donor and recipient require dissimilar antibiotic resistance, suicide vector pST76-A, containing an ampicillin resistance cassette and a temperature-sensitive origin of replication, was integrated into pSYM1 by homologous recombination, resulting in pSYM1-ST76An. Then, recipient strain *E. coli* G4/9 was transformed with pACYC184 conferring it with chloramphenicol resistance. Donor *E. coli* G3/10 and recipient *E. coli* G4/9 were grown in LB medium to an absorbance of A_600_ ∼ 0.5 to 1, each containing the appropriate antibiotic. Cells were pelleted in a bench top centrifuge at 5000 g for 10 min, washed twice with phosphate buffered saline (PBS) and resuspended in LB medium. Donor and recipient were mixed 2∶1, pelleted and resuspended in a small volume of LB medium (∼100 µl). The cell suspension was plated on blood agar (BD) and incubated overnight at 37°C. Bacteria were resuspended in 1 ml LB medium, diluted in PBS and plated on MacConkey agar (BD) containing 25 µg ml^-1^ chloramphenicol and 100 µg ml^-1^ ampicillin (AppliChem). The resulting clones were confirmed by PCR.

### Sequencing and Annotation of pSYM1

Plasmid pSYM1 was discovered as one of six wild-type plasmids in *E. coli* strain G3/10. The genome of the strain was obtained by pyrosequencing with a 454 GS-FLX System (Roche, Basel, Switzerland) (unpublished data). About ninety-five percent of the plasmid sequence was amassed during genome sequencing and assembly. Then, primers for an outwardly directed primer walking strategy were designed (Table 3) until a completely closed plasmid sequence was obtained. Annotation was performed automatically at CeBiTec followed by manual revision using GenDB 2.4 software [36]. BLASTn and BLASTp searches were done using the National Center for Biotechnology Information (NCBI) website [37].

### LOVO and CACO-2 Cell Culture Conditions

The human intestinal epithelial cell lines LOVO [38] and CACO-2 [39] , purchased from DSMZ [40], were grown in RPMI-1640 cell culture medium (Biochrom, Berlin, Germany) supplemented with 10% FCS (Biochrom) and maintained in an atmosphere of 5% CO_2_ at 37°C. Cells reached confluency after 3–4 days and were used consistently within 3–4 days from seeding. Cell cultures were tested routinely and found to be free from mycoplasma contamination.

### In vitro Adherence Assay

An *in vitro* adherence assay was performed according to a modified protocol from Kleta et al. [21]. Briefly, human intestinal epithelial LOVO or CACO-2 cells were seeded in 24-well plates and grown to confluency. Bacterial strains to be tested were grown in LB broth to an absorbance of A_600_ ∼ 0.6, whereas the adherent enteropathogenic *E. coli* strain E2348/69 was grown to A_600_ ∼ 1, each supplemented with the appropriate antibiotic. One ml of each bacterial strain was pelleted, washed with PBS and resuspended in cell culture medium. LOVO and CACO-2 cells were infected with the bacteria to be tested at a multiplicity of infection (MOI) of 100∶1 *E. coli* to host cells. After an incubation period of two hours at 37°C and 5% CO_2_ bacteria were washed away with PBS for three times and host cells were infected with EPEC. Bacteria were allowed to adhere for six hours in total (37°C, 5% CO_2_), while the cell culture medium was replaced after three hours of incubation. Cells were washed with PBS and lysed with 0.01% Triton-X-100 in PBS (Sigma-Aldrich, Munich, Germany). The number of adherent EPEC was determined by plating serial dilutions on LB agar plates containing the appropriate antibiotic. Adherence efficiency in percentage is expressed as adherence of EPEC relative to the adherence without any pre-incubation (negative control), which is set at 100%. All strains were tested in at least three independent experiments.

### Screening of mcsS Presence in Enterobacteria

Gene *mcsS* encoding microcin S was amplified by a multiplex PCR protocol from culture material, using *recA* as an inhibition control. Oligonucleotides were used as indicated in table 3. *E. coli* G3/10 served as positive control. Thirty-eight different *E. coli*, two Shigella and two Salmonella strains were screened. To our knowledge, none of the isolates tested has been sequenced. Strains are common lab strains as well as isolates of human or animal origin as indicated in figure 6.

### Statistical Analysis

Data are expressed as mean±SD. Student’s *t*-test was used to determine the statistical significance. p≤0.05 was considered statistically significant.

### Nucleotide Sequence Accession Number of pSYM1

The pSYM1 plasmid sequence has been deposited in the GenBank sequence database with the accession number JN887338.
